# Your move: The effect of chess on mathematics test scores

**DOI:** 10.1371/journal.pone.0177257

**Published:** 2017-05-11

**Authors:** Michael Rosholm, Mai Bjørnskov Mikkelsen, Kamilla Gumede

**Affiliations:** 1 TrygFonden’s Centre for Child Research, Aarhus University, Aarhus, Denmark; 2 IZA, Bonn, Germany; 3 Department of Psychology and Behavioural Sciences, Aarhus University, Aarhus, Denmark; University of Zurich, SWITZERLAND

## Abstract

We analyse the effect of substituting a weekly mathematics lesson in primary school grades 1–3 with a lesson in mathematics based on chess instruction. We use data from the City of Aarhus in Denmark, combining test score data with a comprehensive data set obtained from administrative registers. We use two different methodological approaches to identify and estimate treatment effects and we tend to find positive effects, indicating that knowledge acquired through chess play can be transferred to the domain of mathematics. We also find larger impacts for unhappy children and children who are bored in school, perhaps because chess instruction facilitates learning by providing an alternative approach to mathematics for these children. The results are encouraging and suggest that chess may be an important and effective tool for improving mathematical capacity in young students.

## 1. Introduction

The costs of primary and lower secondary schooling in Denmark are among the highest in OECD. This is in part due to large amounts being spent on children in special needs education; about one third of all the costs of primary and lower secondary education goes to special needs education, aimed at both students with learning disabilities but also students with behavioural problems. In spite of high levels of spending, Denmark is ‘average’ in the OECD according to cross-country data from the PISA studies [1]. In particular, the Danish school system seems to have problems aiding learning in the weakest students and the very best students. Since mathematics is particularly important, e.g. in terms of minimum requirements for gaining access to post-secondary education, political as well as research interest has been on interventions that may improve students’ mathematical abilities and reasoning.

In the international literature, focus has recently switched towards non-cognitive factors when seeking explanations for educational failure. Factors such as emotions, personality, behavioural problems and lack of self-control are increasingly brought forth as explanations [2–11]. A recent longitudinal study [12] is of particular interest in this regard. They examined data from 4.600 middle-school students from 24 different schools and found that, although prior grades and standardized achievement were the strongest predictors of high school grade point average (GPA), psychosocial and behavioural factors (e.g. “Skipped class”, “Academic discipline” and “Commitment to school”) were also significant predictors of GPA. The results suggest that non-cognitive factors should not be neglected if the purpose is to enhance academic performance. The evidence presented above points to the importance of both cognitive and non-cognitive factors in achieving educational success.

Teaching children chess may help them acquire cognitive skills, including math skills, directly, as well as indirectly through non-cognitive factors.

In this study, we investigate whether chess instruction leads to improvements in math test scores of primary school children in Denmark relative to a comparison group receiving ordinary math lectures. Specifically, we compare substituting a ‘normal’ weekly math lecture with a math lecture based on chess instruction in a fairly large sample of Danish schoolchildren. We control extensively for parental and child background using a rich data set based on administrative registers. Bart [13] argues that to understand and evaluate chess positions, you must take into account the different mobility patterns of the pieces, requiring fluid intelligence and concentration capacity. You then have to formulate and evaluate possible moves, requiring executive functioning, pattern recognition, and critical thinking. He goes on to argue that for these reasons, chess may lead to cognitive improvements. Moreover, there is a set of rules of conduct during a chess game; you shake hands at the start and end of a game, you sit quietly during the game, you often discuss the game with your opponent or teammates afterwards, hence teaching you how to learn from your mistakes and inspiring and illustrating the potential gains from learning, see e.g. Ericsson *et al*. [14] on the importance of deliberate practice. As such, chess requires both cognitive abilities (attention, perception, information processing, memory and problem solving) and non-cognitive skills (patience, discipline, self-control and social skills). Strengthening these skill sets through chess may prove beneficial for children’s academic performance.

Inherent to this suggestive postulate is the belief that abilities acquired through chess can be transferred to other domains. Whether this belief is well founded will be addressed in the following section on the existing evidence of the link between chess and academic performance.

## 2. Chess and academic performance

In this section, we will first discuss the theoretical end empirical literature on transfer of abilities. Secondly, we will explore the evidence of transfer from chess to other domains with special attention to the link between chess and mathematical performance. We will conclude with a brief section on potential indirect channels through which chess play can influence mathematical performance.

### Transfer of abilities

*Transfer* is a broad term used to denote generalization of abilities acquired within one domain to other domains [15]. In the context of the current study, at least two theoretical distinctions related to transfer are worth mentioning. The first concerns the transfer context and pertains to similarity of domains between which transfer is to occur. “*Near-transfer*” refers to transfer of knowledge between highly similar domains, while “*far-transfer*” relates to transfer of knowledge between very heterogeneous domains [16]. Generally, the available empirical evidence suggests that far-transfer is very rare [17–19], while newer studies give some scientific merit to the existence of near-transfer [20–22]. The degree of similarity between domains thus appears to be important for the transfer of knowledge.

A second relevant distinction is made by Detterman [23] and relates to the content that is transferred. Detterman distinguishes between *“specific”* and *“non-specific”* transfer, where the former refers to transfer of specific, concrete content, while the latter concerns transfer of concepts, principles and general ideas. Empirical evidence suggest that highly specific knowledge is less likely to be transferred to new domains, especially if two domains do not share common features [24–26]. Specificity of the acquired abilities can therefore be seen as another factor influencing the transfer of knowledge and abilities.

In the context of chess and transfer of skills acquired through chess to other domains, it is evident that far transfer is implied [27]. The domains of chess and mathematics seem to be contextually more dissimilar than similar, thus impeding transfer of abilities between the two. On the other hand, chess cultivates highly non-specific abilities (e.g. problem solving, persistence, focusing, self-control, working memory etc.), which can be considered relevant for successful performance within academic domains in general [28]. Alongside the non-specific abilities, chess also promotes more specific abilities such as understanding of numerical and spatial relationships and approaching quantity-based problems, which relate more directly to the mathematical domain [27]. In summary, the available theoretical and empirical evidence on general transfer of abilities suggests that there could be both hindrances and supportive factors influencing the transfer of abilities from chess to academic performance, in general, and to mathematics in particular. In the following section, we will explore the available evidence specifically related to transfer of chess skills.

### The transfer of chess skills

There is a large body of evidence pertaining to the cognitive and psychological underpinnings of playing chess. Chess players have been found to be more intelligent, more open and extroverted and to have better spatial abilities than the general population [29–31]; however, this may be due to self-selection and may thus not be evidence of transfer.

Berkman [32] explicitly discusses the link between chess and mathematics and argues that chess promotes higher-order thinking skills, and that the analysis of chess positions has much in common with problem solving in mathematics. It works with concepts as correlation, it uses the coordinate system, geometric concepts such as rows and columns (called ranks and files in chess), diagonals and orthogonals, and it requires continuous calculation. It also develops visual memory, attention span (concentration), spatial reasoning skills, capacity to predict and anticipate consequences, critical thinking, self-confidence, self-respect, and problem solving skills (see also [33–34]).

A recent meta-analysis conducted by Sala and Gobet [27] suggests that skills acquired through chess instructions do indeed transfer to academic domains. The authors reviewed 24 studies with 2788 young people in chess conditions and 2433 controls, and found a moderate effect of chess based instruction on overall cognitive and academic ability (g = 0.34). The results further indicated that the effect size for mathematics (g = 0.38) was larger than for reading (g = 0.25). Although the differences between the domains of mathematics and chess are plenty, they can still be considered more similar than the domains of chess and reading. Thus, the results are consistent with the empirical evidence on near- and far-transfer, speaking to the importance of domain-similarity for transfer of knowledge and abilities.

Both Gobet & Campitelli [35] and Bart [13] review empirical evidence on the relation between chess and several educational outcomes. Gobet & Campitelli [35] base their review on seven studies, of which only two are published in peer-reviewed journals and conclude that it is still an open question whether chess instruction improves learning in other areas than chess.

Bart [13] summarizes a few more recent studies and concludes more positively, that chess instruction has positive effects on scholastic achievements. The included studies suggest a positive causal link from chess instruction to mathematics achievement and non-verbal cognitive ability, intelligence and problem solving ability, cognitive ability and math test scores, math test scores for low ability students with IQs in the ranges of 70–85, math test scores (and end-of-year grades) for pupils with special education needs, and to non-verbal intelligence for students at risk of academic failure. However, many of these results are based on studies with small sample sizes.

A few studies with more appropriate sample sizes have been conducted recently. Işıkgöz [36] examined end-year math scores of 274 pupils (137 playing chess) in five secondary schools. He finds a significant difference in end-year math scores in favour of the chess-playing students. It is, however, unclear how much time the treatment group spent playing chess and whether the control group received a similar amount of regular math instruction. Moreover, the establishment of causality is not convincing, given that there was no randomization, quasi-random variation, or pre-intervention test scores.

Trinchero & Sala [37] conducted an impressive study of 931 third, fourth and fifth graders from 20 different schools. The students were randomly assigned to either chess training performed by chess instructors, chess training performed by school teachers, or a control group. Chess instructors were provided with specific instructions on how to teach chess problem-solving heuristics to the children, while school teachers were not. Tests of mathematical problem-solving ability before and after the 6-month intervention indicated that chess instructions only improves problem-solving ability if it conveys problem-solving heuristics to pupils. The authors suggest that chess instructors may have facilitated broader problem solving abilities and flexible thinking during instructions while schoolteachers may have been more focused on conveying basic rules.

As the above evidence suggests, several studies have found chess to improve academic performance in general and mathematical capacity, in particular. The study by Trinchero & Sala [37] is unquestionably the most impressive study so far, as it employs a design of a higher quality than previous studies and is more adequately powered. Moreover, results from the study point to an effect of chess on math scores providing evidence that abilities and knowledge acquired during chess play can be transferred to the domain of mathematics. The results further indicate that a possible mechanism behind the improved math abilities may be transfer of non-specific knowledge (i.e. problem-solving and flexible thinking).

### Potential indirect channels; non-cognitive factors

As the previous section suggests, chess instruction appears to have direct impacts on the capacity for learning mathematics, but there may also be indirect impacts operating through non-cognitive factors. One such factor is affect. Affective states are central components of engagement and motivation and thereby an essential driving force for successful learning [38]. In the following, we briefly review empirical evidence on two affective states that are particularly relevant for the current study: Boredom and happiness.

Recent educational research has seen a surge of interest in affective states and how they relate to academic performance in young students. Boredom is an affective state of particular relevance in academic settings, as most students report being bored in class occasionally [39]. Boredom is often defined as an unpleasant affective state associated with lack of interest and difficulties attending to a task [40]. The Control-Value theory proposed by Pekrun and colleagues is a prevalent framework for understanding affect in educational settings and suggests that affective states are determined by how controllable an academic activity is perceived to be and the subjective value assigned to it [2, 41]. Within this theory, boredom occurs when students do not value an activity because they perceive it to be too demanding, too easy or irrelevant. Empirical evidence concerning the relationship between boredom and academic performance in young students is scarce. A recent meta-analysis by Tze *et al*. [39] assessed 29 studies involving 19.052 secondary and tertiary students and found a moderate negative relationship between boredom and students’ academic performance (r = -.24). This finding is similar to findings by a recent study conducted with 557 young students, where boredom was found to be significantly negatively related to graded performance [42]. Specifically pertaining to the effect of boredom on mathematical performance, two recent studies suggest a negative link. Lichtenfeld *et al*. [43] find boredom to be negatively related to mathematical performance in a sample of 1190 second- and third grade students. Ahmed *et al*. [44] followed 522 grade 7 students over a school year and report findings that suggest a negative effect of boredom on math grades.

Another affective state that could potentially influence the capacity for learning is happiness. Happiness can be defined as a positive, pleasant affective state, and within an educational context, it often arises when a student experiences success or, following the Control-Value theory, when a student values the learning material and perceives he is able to handle it [41]. Like with boredom, only a few studies have investigated the association between happiness and academic performance. Pekrun *et al*. [45] summarized findings from 11 studies of secondary and tertiary students and found that positive emotions (with the exception of relief) predict high academic achievement. Kwon *et al*. [46] studied 417 elementary school students and found that happiness was indirectly associated with academic achievement through academic engagement. Mega *et al*. [47] provide evidence of an indirect relationship between positive emotions and academic performance in a sample of 5.805 undergraduate students. More specifically, the authors found that positive emotions influence academic achievement through facilitation of self-regulated learning and motivation. Only a few studies have investigated the relationship between happiness and math performance. In the previously mentioned studies by Lichtenfeld *et al*. [43] and Ahmed *et al*. [44], results indicated a positive relationship between happiness and math grades and between enjoyment and math grades.

Hence, summarizing, the empirical evidence on the predictive value of boredom and positive emotions (including happiness) for achievement within an educational context is limited. The available evidence do, however, indicate that the two affective states are associated with both academic performance and math ability, thus providing grounds for the premise that chess instruction may affect math abilities indirectly through boredom and happiness.

### Hypotheses

From the literature review, we found evidence that chess instruction may affect mathematics abilities directly as well as indirectly through affective states. Based on existing empirical evidence, we hypothesize that chess instruction leads to improved mathematical abilities and reasoning, especially in problem solving and pattern recognition tasks. We further hypothesize that impacts may be moderated by boredom and/or happiness.

## 3. Methodology

### Participants

The intervention took place in five schools in the City of Aarhus, the 2^nd^ largest city in Denmark. Two of the schools are located in socially deprived sub-urban areas, two are in middle class suburban areas, and the fifth school is located close to downtown Aarhus, in a middle-income area, but with some social housing. 482 children from selected classes (grades 1 to 3) were chosen to participate in the study. The classes selected for participation were not randomly assigned, but rather assigned to treatment or control conditions by the school principal. The criteria for assignment of classes to treatment and control groups were unclear, but subsequent interviews suggest that they were close to random, or at least based on factors not immediately related to the children’s capacity for learning. This is also confirmed in the Results—Descriptive statistics—section. Table 1 below shows the distribution of participants across (anonymized) schools.

**Table 1:** The distribution of treatments and controls across schools.

Schools	Treatment group Students (classes)	Control group Students (classes)
**School 1 (middle sub-urban)**	91 (4)	39 (2)
**School 2 (downtown)**	14 (1)	15 (1)
**School 3 (middle class sub-urban)**	84 (5)	52 (3)
**School 4 (socially deprived sub-urban)**	110 (5)	33 (3)
**School 5 (socially deprived sub-urban)**	24 (2)	20 (2)
**Total**	323	159

### Procedure

**Fig 1:**
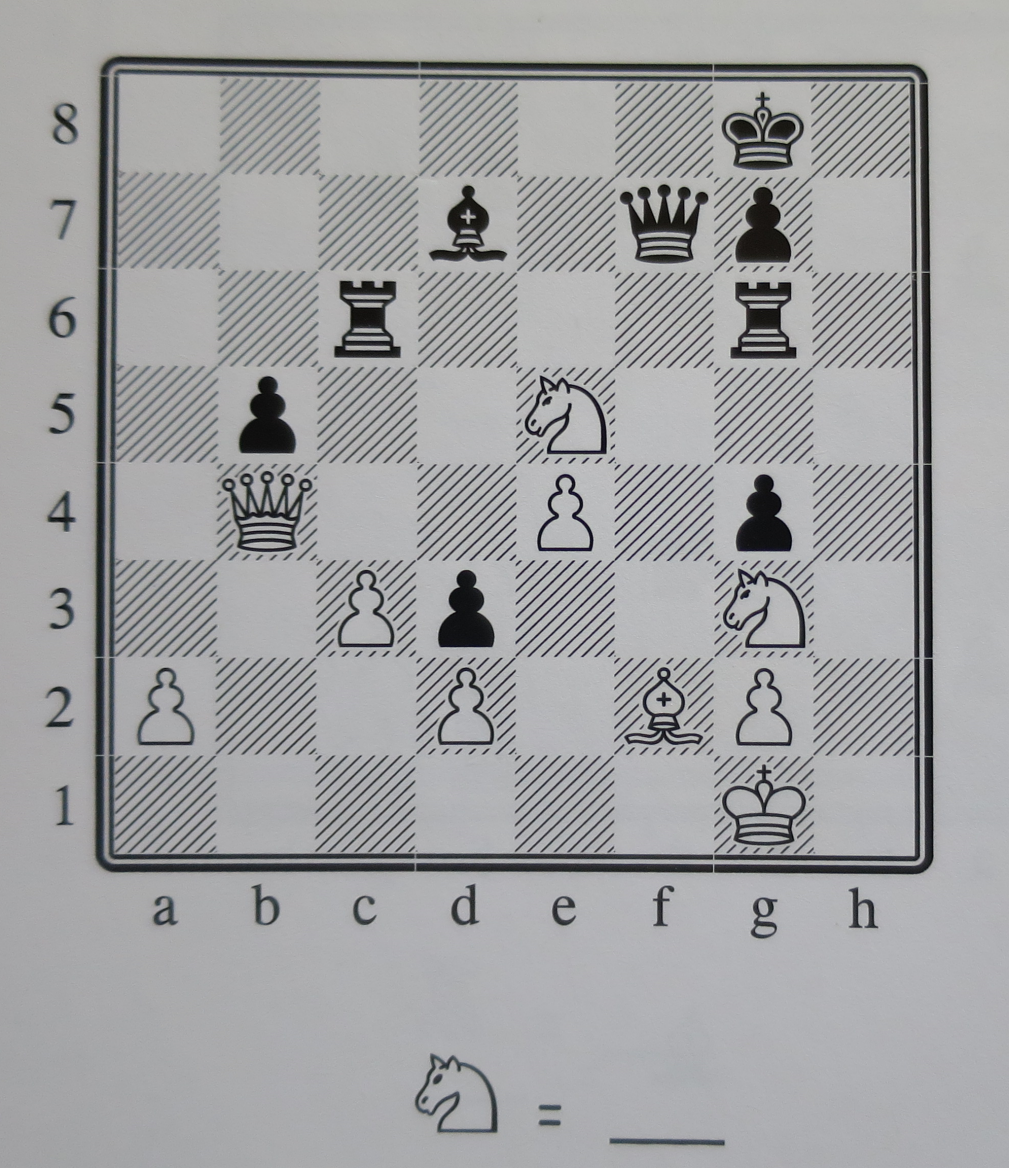
A typical chess exercise from the book used for chess instruction.

Of the 482 students participating in the current study, 323 had one (in four) weekly mathematics lecture (45 minutes) replaced with chess instruction for the spring semester of 2013 and the first half of the fall semester (until mid-October). 159 children in the control group did not participate in the program but instead received four weekly math lectures (treatment as usual). In this sense, our design is different from the studies mentioned previously, as they all consider the treatment condition (chess instruction) as an extra activity, rather than a replacement activity (for mathematics). Therefore, we would expect to find smaller effect sizes than the reviewed studies. A dedicated mathematics teacher, who is also a club chess player, performed the teaching during treatment lectures in all five schools. The regular mathematics teacher was occasionally present in the classroom during chess lectures and would participate in playing if there was an odd number of students present in the classroom but would not otherwise take part in teaching during these chess lectures. Lectures consisted partly of instruction on the movements of chess pieces, and partly of practical chess playing exercises. The chess lectures were based on a book developed by the Danish School Chess Association (Dansk Skoleskak) called Chess+Math (Skak+Mat). Fig 1 below depicts a typical exercise from the book. The instruction for the exercise is (translated from Danish): *“How many pieces can the knight take*? *Write your answer on the line below”*.

The intervention was originally intended to end before the summer holidays of 2013 (end of June, 2013), but due to a lock-out of the teachers as the result of a conflict in relation to central negotiations, all public schools were closed during most of April 2013. Hence, it was decided to extend the intervention into the next school year and end it by mid-October of 2013. In total, the pupils thus had around 30 math lectures replaced with a lecture of chess instruction. The total number of lectures was not precisely registered, but there were very few cancellations.

Pupils in treatment as well as control classes in the same schools were administered a mathematics test immediately before the start of the intervention. Students were then administered a post-intervention test in November-December 2013, taking a test which was a grade level higher than the pre-intervention test. The information on the test-scores and treatment status was merged with a large database collected by TrygFonden’s Centre for Child Research at Aarhus University based on administrative registers in Statistics Denmark and is physically located in Statistics Denmark. It contains information on parental socioeconomic background, immigrant status, gender, a few schooling outcomes (including days of school absence) and is used in order to control as comprehensively as possible for children’s characteristics and parental background.

In addition to the information from the administrative registers, two questions were administered to the children during the mathematics test (both the pre- and post-intervention tests); one on happiness in general and one on whether they were bored in school. There were three possible answers (unhappy to very happy; often bored to never bored), which have both been coded from minus 1 to 1.

### Measures

The mathematics tests used to assess mathematical abilities and reasoning pre- and post-intervention were designed to test students in calculation and geometry, pattern recognition (numbers and shapes), and basic problem solving. The calculation and geometry components are addition, subtraction, multiplication, division, basic equations (e.g. 4+x = 9), and counting, say, the number of triangles in a cloud of shapes. Patten recognition is both number sequences (e.g. 1-2-4-8-16-_-_, 1-4-9-16-25 _ _, or 1-1-2-3-5-_-_) and finding shapes that fit into a sequence of shapes and colours. Finally, problem-solving skills were assessed with the following question *“Mette arrives at a bridge*. *A troll is guarding it*. *He says ‘if you want to pass the bridge and come back*, *I will double the amount of money you have in your pocket*. *Afterwards you have to give me 8 kroner’*. *Mette passes and gives the troll 8 kroner*. *Now Mette has no more money left in her pocket*. *How much did she have in her pocket when she arrived at the bridge*?*”* The tests were rather comprehensive in calculation and pattern recognition, while there was only one problem solving exercise. For this reason we suspect that we may have difficulties assessing treatment effects precisely in problem solving.

The tests varied slightly from grade to grade. The test for 2^nd^ grade had 4 questions on calculation giving a total of 14 points, the questions for pattern recognition gave a total of 10 points, and the problem solving exercise gave either 0 or 4 points. For grades 1, 3 and 4, it was quite similar in terms of weighting.

The test scores were subsequently standardized within grade levels and by whether it was a pre-treatment or post-treatment test, subtracting the average within the group and dividing by the standard deviation of the pooled (treatment and control group) data. Thus, within each test period and grade level, standardized test scores have mean zero and standard deviation 1. This implies that the estimated impacts are directly interpretable as effect sizes.

### Statistics

Since the treatment and control groups were not randomly assigned, the difference in raw post-treatment test-scores between treatment and control groups cannot be given a causal interpretation. The raw difference-in-differences estimator (the difference in the change in test scores from pre to post treatment) can be interpreted causally under the assumption that, in the absence of treatment, the improvement in test scores would be the same in the two groups (the parallel trends assumption). In order to strengthen the causal interpretation and to try to improve statistical power, we would nevertheless prefer to control for school factors, parental and child background variables.

Denote by *Y*_*it*_ the outcome of interest (e.g. the math test score) for individual *i* at time *t*. Let *t = 1* denote the post-intervention period, and let *t = 0* denote the pre-intervention period. Hence, *Y*_*i0*_ denotes the pre-intervention test score of student *i*, and *Y*_*i1*_ the post-intervention test score. Let *D*_*i*_ denote the treatment status of student *i* (D = 1 denotes treatment, 0 control), and let *X*_*i0*_ denote a set of background characteristics (specifically, the variables reported in Table 2 below, except for the pre-intervention test score). Finally, let *S*_*i*_ denote a set of school indicators (fixed effects).

**Table 2:** Descriptive statistics, background variables.

Variable	Treatment group	Control group	P value
**Standardized pre-intervention test-score**	0.00	0.05	0.64
**Boy**	0.54	0.50	0.41
**Girl**	0.46	0.50	0.41
**Age**	9.57	9.45	0.14
**1^st^ or 2^nd^ generation immigrant**	0.28	0.25	0.36
**Days of school absence 2012**	9.21	9.94	0.39
**Grade 1**	0.19	0.31	<0.01
**Grade 2**	0.45	0.33	0.01
**Grade 3**	0.36	0.36	0.94
**# siblings**	1.46	1.53	0.44
**Happiness (-1;0;1)**	0.38	0.41	0.60
**Not bored (-1;0;1)**	0.49	0.43	<0.01
**Mother present in household**	0.99	0.97	0.30
**Age of mother**	40.53	40.42	0.82
**Mother lower secondary school**	0.42	0.41	0.80
**Mother high school**	0.07	0.08	0.67
**Mother vocational education**	0.27	0.22	0.24
**Mother short academic education**	0.05	0.06	0.44
**Mother medium academic education**	0.07	0.08	0.67
**Mother masters education or more**	0.11	0.14	0.29
**Mother’s education missing**	0.02	0.02	0.68
**Mother’s average ann. earnings past 3 years, DKK**	195,276	188,578	0.64
**Mother not working 2011**	0,29	0,30	0.92
**Father present in household**	0.79	0.76	0.53
**Age of father**	43.28	42.28	0.17
**Father lower secondary school**	0.19	0.15	0.27
**Father high school**	0.05	0.04	0.47
**Father vocational education**	0.30	0.30	0.97
**Father short academic education**	0.08	0.10	0.39
**Father medium academic education**	0.14	0.13	0.68
**Father masters education or more**	0.17	0.18	0.74
**Father’s education missing**	0.07	0.11	0.18
**Father’s average ann. earnings past 3 years, DKK**	278,564	284,405	0.78
**Father not working 2011**	0.19	0.20	0.68
**N**	323	159	

Our first model of interest then seeks to explain the post-treatment standardized test score with the pre-treatment standardized test score, a treatment indicator, a set of explanatory background characteristics, a set of school fixed effects, and a residual error term:

$$Y_{i1}=\mu +\alpha Y_{i0}+\gamma D_{i}+\beta X_{i0}+\delta S_{i}+\varepsilon_{i}$$ (1)

The treatment effect, *γ*, is estimated consistently under the assumption of conditional independence (see e.g. [48]), that is, conditional on the included parental and background variables, and school fixed effects, the expected value of *Y*_*i*1_ is the same in the treatment and control group. This is also known as the ‘unconfoundedness’ or the ‘selection on observables’ assumption. This assumption requires access to a large set of potentially confounding variables. Our administrative register data with a large set of background information on children as well as their parents is a great strength in this sense.

An alternative formulation would be a ‘learning’ model:

$$Y_{i1}-Y_{i0}=\tau +\theta D_{i}+\vartheta X_{i0}+\pi S_{i}+\rho_{i}$$ (2)

This model is slightly different from (1) in the sense that it is the change in the test scores, that is, the speed of acquisition of mathematics capability, that is the dependent variable. The learning speed is in this specification a function of treatment, the included parental and background characteristics, and school fixed effects. In this specification the treatment effect is identified under a weaker condition than conditional independence; namely, the parallel trends assumption discussed above. Any unobserved individual specific confounding variables affecting *Y*_*i1*_ and *Y*_*i0*_ directly are ‘differenced out’ of the estimation. Heckman *et al*. [49] argue that a difference-in-differences strategy, as the one specified in Eq (2), is often the best strategy for estimating a causal effect, when assignment to the treatment is not entirely random.

In the results section, results for both specifications are reported, as they both have some merit and rely on different identifying assumptions, for which reason they serve as a robustness test of the results. As we have no prior reason for strongly believing that replacing mathematics with chess instruction should improve math test scores, we choose to use two sided test statistics. We have chosen the conventional 5% significance level.

## 4. Results

### Descriptive statistics

Table 2 compares averages for demographic information retrieved for the treated children and for children in the control group.

Despite the fact that the distribution across grades differ quite a bit, with significantly more treated children in grade 2 and significantly fewer in grade 1, the children (and their parents) do not differ as much. Only one of the differences, except grade levels, is statistically significant, namely, the treatment group is slightly less bored in school *ex ante* The control group appears to have slightly higher test scores *ex ante*, but the difference is small and is not statistically significant. Moreover, mothers of children in the control group appear to be slightly better educated, although none of these differences are significant either. Still, given the potential non-random nature of the assignment to treatment and control conditions, it is important to control for these background factors. In this respect, access to *ex ante* test scores is a large advantage, as it allows us to exploit a difference-in-differences strategy for identifying the causal impact of the intervention.

### Outcomes

Table 3 shows the average standardized post-intervention test-scores and the average change in standardized test scores.

**Table 3:** Average standardized outcomes.

Variable	Treatment group	Control group	P value
**Standardized post-treatment test score**	0.05	-0.09	0.16
**Change in standardized test score**	0.05	-0.13	0.04

The averages in the two post-treatment test scores differ by 0.14 in favour of the treatment group, but the difference is not significantly different. The raw average difference-in-differences is 0.18 in favour of the treatment group and is statistically significant (at the chosen 95% level).

### The effect of chess instruction on math scores

In this section, we present the results of the two models introduced in the methodology section. First, Table 4 shows estimation results, where the post-intervention test score is the dependent variable (corresponding to Eq 1 above). We show three different models; one where we include the indicator for having received a weekly chess-lesson instead of a weekly math lesson and the pre-intervention test score (model 1). In model we add a set of child characteristics that showed significant group differences in Table 2, and finally model 3 also adds school fixed effects.

**Table 4:** Estimation results, post intervention test-scores.

	B	SE(B)	t	Sig. (p)	F	R-squared
**Model 1:**					116	0.33
**Pre-intervention test score**	**0.57**	0.04	15.13	0.00		
**chess dummy**	**0.16**	0.08	2.03	0.04		
**Model 2:**					46	0.33
**Pre-intervention test score**	**0.57**	0.04	15.10	0.00		
**Grade 2**	-0.02	0.10	0.18	0.86		
**Grade 3**	0.02	0.10	0.15	0.88		
**Not bored**	0.05	0.07	0.73	0.47		
**Chess dummy**	0.16	0.08	1.93	0.06		
**Model 3:**					30	0.37
**Pre-intervention test score**	**0.53**	0.04	13.88	0.00		
**Grade 2**	-0.21	0.11	1.80	0.07		
**Grade 3**	-0.16	0.12	1.34	0.18		
**Not bored**	0.07	0.07	1.10	0.27		
**School 1**	**-0.53**	0.18	2.91	0.00		
**School 2**	0.11	0.10	1.10	0.27		
**School 3**	**0.26**	0.11	2.46	0.01		
**School 4**	**-0.33**	0.15	2.18	0.03		
**Chess dummy**	0.10	0.08	1.28	0.20		

Note: **Bold numbers** imply statistical significance at the 95% level.

First, note that the pre-intervention test-score explains about one-third of the variation in the post-intervention test scores. The estimated effect size is statistically significant once we control for pre-intervention test scores, but the statistical significance disappears again when additional control variables are added. The effect sizes are 0.10–0.16.

Table 5 shows the effects from the learning model (Eq 2). When only the indicator for receiving chess instruction is included, the effect is statistically significant, but the impact declines slightly once additional characteristics are included, rendering it insignificant at the 95% level. The effect sizes are 0.16–0.18 in this model.

**Table 5:** Estimation results, change in test-scores.

	B	SE(B)	t	Sig. (p)	F	R-squared
**Model 1:**					4	0.01
**Chess dummy**	**0.18**	0.09	2.03	0.04		
**Model 2:**					1	0.01
**Grade 2**	-0.02	0.11	0.21	0.84		
**Grade 3**	0.01	0.11	0.11	0.91		
**Not bored**	0.06	0.08	0.83	0.41		
**Chess dummy**	0.18	0.09	1.91	0.06		
**Model 3:**					2	0.03
**Grade 2**	-0.19	0.13	1.43	0.15		
**Grade 3**	-0.14	0.13	1.08	0.28		
**Not bored**	0.06	0.08	0.75	0.46		
**School 1**	-0.30	0.21	1.40	0.16		
**School 2**	0.13	0.12	1.08	0.28		
**School 3**	**0.25**	0.12	2.06	0.04		
**School 4**	0.05	0.17	0.28	0.78		
**Chess dummy**	0.16	0.09	1.71	0.09		

Note: **Bold numbers** imply statistical significance at the 95% level.

For completeness, we have investigated sub group impacts by gender, immigrant status, and pre-intervention test scores. We found a significant positive effect for native Danes and a significantly lower (in fact, negative) effect for immigrant children and a tendency to larger effects for boys than girls. These results are not pursued further in this study.

#### Effect by grade level

Table 6 presents the effects of chess instruction separately by grade level. The chess dummy reports the effect for grade 3, while the two interaction terms reflect the deviation from this effect in grades 1 and 2. There are no significant differences—nor are there significant impacts—across grades, but this is probably due to the lack of sufficient statistical power (see *Limitations)*.

**Table 6:** Impact estimates by grade.

	Post-intervention test score effects	Change in test-score effects
**Chess dummy**	0.14 (0.13)	0.27 (0.15)
**Chess dummy x Grade 1**	-0.01 (0.20)	-0.03 (0.23)
**Chess dummy x Grade 2**	-0.11 (0.19)	-0.27 (0.21)

Note: Results are from model 3 in Tables 4 and 5. **Bold numbers** imply statistical significance at the 95% level.

#### Effects on separate domains

In Table 7, we report effects on the normalized test scores separately by domains; pattern recognition, problem solving, and calculation. There is a significant positive impact only on pattern recognition, while there are no effects on problem solving. When we split the exercizes on pattern recognition into numerical and figural exercizes, we find that the impact stems almost entirely from the numerical exercizes.

**Table 7:** Impacts by different math domains.

	Post-intervention test score effects	Change in test-score effects
**Pattern recognition**	**0.47** (0.17)	0.46 (0.24)
**Numerical patt. recog.**	**0.29** (0.09)	0.18 (0.10)
**Figural patt. recog.**	0.10 (0.10)	0.08 (0.10)
**Problem solving**	-0.12 (0.16)	0.07 (0.21)
**Calculation**	0.11 (0.45)	0.21 (0.46)

Note: Results are from model 3 in Tables 4 and 5. **Bold numbers** imply statistical significance at the 95% level.

#### Effects by happiness

In Table 8, we have interacted the treatment indicator with the variable on happiness. First, we find that happier children tend to perform better in mathematics. In addition, we find that the impact of chess instruction is larger for the students who are less happy at the outset and a tendency for these effects to disappear for the more happy children.

**Table 8:** Impacts by happiness.

	Post-intervention test score effects	Change in test-score effects
**Chess dummy**	**0.21** (0.10)	**0.24** (0.11)
**Happy**	0.21 (0.12)	0.09 (0.14)
**Chess dummy x Happy**	-0.25 (0.14)	-0.20 (0.17)

Note: Results are from model 3 in Tables 4 and 5. **Bold numbers** imply statistical significance at the 95% level.

#### Effects by boredom

In Table 9, the treatment indicator is interacted with the question on whether or not the child is bored in school. Those who are never bored are better at mathematics and tend to learn faster. However, being bored is associated with a significant and fairly large treatment effect; for the most bored (not bored = -1), the treatment effect is 0.56 (0.27+0.29) in the learning model—and statistically significant. Those who are never bored (not bored = 1) do not experience any additional gains from the chess instruction.

**Table 9:** Impacts by boredom.

	Post-intervention test score effects	Change in test-score effects
**Chess dummy**	**0.20** (0.10)	**0.27** (0.11)
**Not bored**	**0.22** (0.10)	0.23 (0.12)
**Chess dummy x not bored**	-0.25 (0.13)	-0.29 (0.15)

Note: Results are from model 3 in Tables 4 and 5. **Bold numbers** imply statistical significance at the 95% level.

We did not find any evidence of impacts on neither happiness, nor boredom, or on days of school absence, neither during nor after the end of the intervention period. Nor did we find any differential impacts on test scores by school absence.

## 5. Discussion

We investigated how chess based mathematics instruction affects mathematics test scores relative to a comparison group receiving ordinary lessons in mathematics. We control extensively for parental and child background, as well as school fixed effects, using a rich data set based on administrative registers.

We find that, on average, replacing one (in four) weekly math lecture with instruction based on chess learning material, during almost three quarters of a school year in grades 1–3 in primary school, leads to an improvement in subsequent math test scores of around 0.10–0.18 standard deviations. This is smaller than what others have found (see e.g. Sala & Gobet [27] who report an average effect size of 0.38 for mathematics), but in those studies, the treatment was incremental to the treatment as usual, while the treatment we analysed in the current study replaced normal math lectures.

According to Lipsey *et al*. [50], a student progresses during a standard school year around 0.5 units of a standard deviation in mathematics in grade 3 and around 1 standard deviation in grade 1. Hence, the estimated effect sizes we find correspond to around one-third of a school year of additional learning of mathematics in grade 3 and one-sixth of a school year in grade 1. This is quite impressive and suggests that the children do acquire capabilities during these lectures, which they can use more generally in school although this was not possible to explore in the present study.

Our findings are thus broadly in line with recent studies on the effect of chess instructions on mathematical ability and reasoning [36–37] and suggest that transfer of abilities between the two domains is possible.

The fact that the treatment group did not receive extra mathematical training and still evidenced a significantly larger improvement on mathematics test compared to the control group suggests that students learned something during chess lessons, that enabled them to better understand mathematics. We cannot say much about the mechanisms behind these results, since children were only tested in mathematics, but the literature study suggested some mechanisms regarding what chess does; there may be a direct effect on mathematics ability, which could manifest itself in improved pattern recognition and problem solving abilities. We did find impacts on pattern recognition, but contrary to recent research [36, 37] and our hypothesis, we did not find impacts on problem solving abilities. This may be due to the fact that only one problem-solving question was included in the math test. Pattern recognition can be considered a broad, non-specific cognitive skill, applicable to several academic domains [51], and our findings thus support the contention that chess promotes acquisition of non-specific abilities relevant for successful learning.

We also found that impacts were driven by children who were not very happy and by those who were occasionally or always bored in school. These results imply that children who are never bored in school and always very happy do not gain anything extra from participating in chess instruction, while unhappy and bored children experience considerable treatment effects. In addition, our results confirm the reviewed studies on affect and educational performance, where happiness has been related to increased academic performance and boredom to the opposite. A crucial methodological difference between the current study and previous studies of the role of affect for educational attainment is the time of measurement. In the current study, we measured the affective states before and after the intervention, while the reviewed studies were primarily cross-sectional and non-experimental. This has important implication for interpretation of our results, as we can only speculate on what affective states the students experienced during chess instructions. In accordance with the Control- Value theory, our results could indicate that chess instructions may have offered the initially bored and unhappy students a way of approaching math that included an increased sense of control and value and thus facilitated learning. Concordantly, the students who were not bored and not unhappy may have perceived the regular math lectures as valuable and the regular material as controllable. As such, the regular math lectures may already have provided an optimal learning environment for these children, and hence they did not derive any additional benefits from chess instructions. Within the control-value framework, this interpretation of our results should imply decreased boredom and increased happiness for the bored and unhappy students following the intervention. Our findings, however, does not support this prediction as we found no significant change in neither happiness nor boredom for the bored and unhappy students from pre-intervention to post-intervention. Although this finding could warrant an alternative theoretical framework, it is also possible that the administered questions regarding happiness and boredom were not sensitive enough to capture subtle changes in the affective states.

The available data does not enable us to conduct a full-blown cost benefit analysis. The costs consisted of learning materials. An additional teacher was used in this intervention, but if the intervention were to be scaled up, rather than costs of extra teachers, there would be costs of training mathematics teachers to use the new learning material. The total costs per pupil in the treatment group was DKK 1857, corresponding to approximately US$ 277. This implies an effect size of 0.36–0.65 per $1000 invested, which is very high when compared to similar statistics for selected US interventions ([52], Fig 6). The benefits are difficult to assess, as we do not know how primary school mathematics improvements affect later life outcomes. Joensen & Nielsen [53] find that taking high-level mathematics in high school causally leads to higher earnings in later life, so to the extent that primary school mathematics improvements are permanent, the long-term gains may be considerable. The argument of dynamic complementarity (cf. [54]) further suggests that early interventions are effective in general, partly because they also make later learning investments more effective; learning begets learning. In this sense, a chess intervention in primary school may have considerable long-term impacts.

### Limitations

Ideally, a randomized trial would have been preferred for estimating the impact of chess instruction, but this was not an option in the present pilot project, so we had to employ alternative statistical strategies for identifying the causal effects of chess instruction. Heckman *et al*. [49] argue that the difference-in-difference strategy used in this study is often the best strategy when assignment to treatment is not random.

The study has a few design flaws compromising its external validity; first, only one teacher was involved in teaching the chess curriculum, and therefore, we cannot be certain of finding similar effects if the intervention were to be scaled up and additional teachers employed. Second, two teachers were present in the classroom during chess lectures, implying that an effect could also be caused by the additional teacher (a two-teacher effect). Andersen *et al*. [55] find positive but smaller effects than ours of a considerably more intensive two-teacher intervention in Danish classrooms, suggesting that a two-teacher effect is not the most likely causal mechanism behind our results.

Finally, despite a comparatively large sample size, the study was underpowered, implying that we were unlikely to find significant impacts. There were 34 different classes involved in the intervention, implying that with an R-squared of 0.37 (as we find in the best of cases) we would have minimum detectable effect sizes around 0.23 with a power of 0.8 and a chosen significance level of 0.95.

Hence, we prefer to think of the project as a pilot study or a demonstration project on the potential beneficial effects of chess instruction. Based on the results obtained here, we are planning a properly designed randomized trial.

## 6. Conclusion

We found that replacing a weekly lecture of traditional mathematics with one based on chess instruction tended to increase subsequent results in math test scores. This consistent with recent research of the impact of chess playing on mathematical abilities and reasoning and suggests that transfer of abilities and knowledge between the two domains is possible. Subgroup analyses revealed that the effect was limited to children who were bored and unhappy while no effect was found for happy children who were not bored. This could indicate an indirect effect of chess instruction on math through reduced boredom and increased happiness. In conclusion, the study demonstrated the potential beneficial effects of chess instructions but further research is warranted.
